# Self-Evaluation of Anxiety in Dental Students

**DOI:** 10.1155/2019/6436750

**Published:** 2019-12-28

**Authors:** Karolina Gerreth, Joanna Chlapowska, Katarzyna Lewicka-Panczak, Renata Sniatala, Michal Ekkert, Maria Borysewicz-Lewicka

**Affiliations:** ^1^Department of Risk Group Dentistry, Chair of Pediatric Dentistry, Poznan University of Medical Sciences, 70 Bukowska Street, 60-812 Poznan, Poland; ^2^Chair of Social Sciences, Poznan University of Medical Sciences, 79 Dabrowskiego Street, 60-529 Poznan, Poland; ^3^Department of Pediatric Dentistry, Chair of Pediatric Dentistry, Poznan University of Medical Sciences, 70 Bukowska Street, 60-812 Poznan, Poland; ^4^Faculty of Medicine, Katowice School of Technology, 43 Rolna Street, 40-555 Katowice, Poland

## Abstract

**Aim:**

The aim of the study was to analyze anxiety in female and male dental students related to their first procedure performed on a pediatric patient as part of their study curriculum.

**Materials and Methods:**

The study was carried out in eighty-four 3rd year dental students (75.00% females and 25.00% males), aged 22–28 years. The participation in the research was anonymous and voluntary. The study was performed during clinical classes in pediatric dentistry where the students were supposed to perform simple prophylactic or therapeutic procedures on pediatric patients. To assess anxiety, a State-Trait Anxiety Inventory was used prepared by Spielberger et al. based on the American STAI questionnaire of 1970 that is composed of two-parts scales: the X-1 scale to assess anxiety as a state and the X-2 scale to assess anxiety as a trait. For statistical analysis, the Wilcoxon signed-rank test, Pearson's chi-squared test, and Mann–Whitney test as well as Statistica 10 programme were used.

**Results:**

The results obtained from the first and second part of the questionnaire concerning anxiety as a state and as a trait showed high level of anxiety as a state in 51.19% of the students and as a trait in 32.14% and low level in 19.05% and 41.67%, respectively. The obtained results showed minimal and maximal values to be 24 and 71, respectively, for the STAI-1 scale (mean = 40.55), and 24 and 57, respectively, for the STAI-2 scale (mean = 41.75).

**Conclusions:**

The results show that the anxiety level during clinical classes is relatively high in the studied population of students. Preparing the students to cope with stress resulting from treating the patients seems to be of importance. Such programmes should be implemented before the start of practical clinical classes. The acquired knowledge will be useful in further professional career.

## 1. Introduction

The notion of “dental anxiety” is usually related to anxiety felt by dental patients [1]. However, it needs to be remembered that people providing health care, such as dental staff, are also overwhelmed with such emotions, especially at an onset of their professional career. The emotions are invariably at their peak when future dentists are preparing for their professions, i.e., during their studies.

Dentists might experience occupational stress from their interaction with staff and patients, problems in the treatment of patients, fears of litigation from their patients, time pressure, and paper work as well as concerns about the financial viability of their practice or defective equipment [2–4]. It is worth mentioning that financial crisis might also affect the medical professionals' well-being [5]. Economic instability can result in mental and physical consequences like anxiety, depression, or psychological well-being [6]. Moreover, uncertainty of students concerning future work may cause such problems.

Medical education aims are, firstly, to master theory and, secondly, to work with patients practically and perform simple therapeutic procedures. Although this is done under professional supervision of teachers, still certain procedures should be carried out by the undergraduate students themselves [7, 8]. Practical classes in pediatric dentistry clinics pose special difficulties as young patients are special. Moreover, the students need to communicate with their parents or guardians. Students of dentistry are exposed to such difficulties because young patients are often anxious for dental treatment.

Literature data show that students' anxiety is increased by their patients' tension caused by the performed procedure [9]. A negative impact of stress on mental and physical health of the students has also been emphasized [9, 10]. It has been proven that stress affects professional efficacy of the trained person by limiting their concentration, attention, and decision-making skills as well as the patient-doctor rapport [10]. Needless to say, mental stress might cause an abnormal activation of the sympathetic nervous system initiating hormonal cascades [5]. Moreover, the psychological condition might worsen the inflammatory response or increase the levels of blood cortisol. Additionally, an increase in some other chronic diseases prevalence, such as asthma, was explained by work-related stress [5].

Dental profession is considered as one of the most stressing medical specialties [11]. Stress-related and musculo-skeletal diseases are the most common reasons why dentists retire from their profession prematurely [11, 12]. It must be emphasized that prolonged psychological and physical exhaustion might lead to work burnout in the susceptible practitioner [2, 13].

There are many anxiety assessment tests applied in psychological practice, one of them being the STAI questionnaire, i.e., State-Trait Anxiety Inventory introduced by Spielberger et al. [14]. The test is widely applied and may be used on various population groups [15]. Moreover, the STAI is used in the screening of the individual diagnosis and research [15].

The aim of the study was to analyze anxiety in female and male dental students related to their first examination performed on a pediatric patient as a part of their study curriculum.

We suggested the following hypotheses: (1) anxiety level of 3^rd^ year dental students while carrying out procedures in pediatric patients will be high; (2) there will be no difference between anxiety level in females and males.

## 2. Materials and Methods

All eighty-four 3^rd^ year Polish dental students of the Poznan University of Medical Sciences, that studied in the academic year 2011/2012, entered the survey between March and April 2012. The group included 63 females (75.00%) and 21 males (25.00%), aged 22–28 years.

The study was carried out during clinical classes in pediatric dentistry where the students were supposed to perform simple prophylactic or therapeutic procedures on pediatric patients. The subject's participation in the research was anonymous and voluntary. The students were informed that they might refuse to participate or withdraw from the study, at any time. Initially, the survey was fully explained to the students and the instruction was read by a supervising psychologist and pedodontist. Following that, the students filled in the questionnaire individually. Data were collected through paper-and-pen questionnaires in a seminar rooms. All the participants gave full answers in 30 minutes. Finally, all participants completed the questionnaire (response rate = 100%).

To assess anxiety, a Polish version of the State-Trait Anxiety Inventory was used which was prepared and adapted by Spielberger et al. [16] based on the American STAI questionnaire of 1970 [14]. This method enables detection of people with definitely low or definitely high level of anxiety as a constant inner predisposition (trait) and is useful to register changes in anxiety intensity in response to specific external stimuli. The STAI questionnaire is composed of two-parts scales: the X-1 scale to assess anxiety as a state, and the X-2 scale to assess anxiety as a trait. Each of them consists of 20 items to which the examined person responds by checking out one of the 4 categorized answers. To describe their subjective feelings towards a statement, the study participant classifies them in the STAI-1 four-point scale as “not at all” (the value of 1), “somewhat” (2), “moderately so” (3), and “very much so” (4), while in the STAI-2 scale as: “almost never” (the value of 1), “sometimes” (2), “often” (3), and “almost always” (4) [15].

The values obtained in each of the scales range from 20 to 80 points, with the 20–40 range described as a low level of anxiety, 41–60 as moderate anxiety, and 61–80 as a high anxiety.

The results were analyzed in three categories of anxiety as a state: low, normal, and high state of anxiety. For the analysis of anxiety as a trait, similar categories were applied.

Continuous variables were presented as means, SD, min value, and max value while nominal variables were presented as a percentage of subjects in particular categories of STAI (low, normal, and high).

Distribution of continuous variables (STAI-1 and STAI-2) was tested by means of Shapiro–Wilk test. Since the data were not normally distributed, the differences between them were assessed with nonparametric Wilcoxon matched-pair test.

The difference test between two proportions was used to check the differences between the percentage of women and men for each level of anxiety, both for anxiety as a state and anxiety as a trait. This test was also used to compare the percentage of people with different levels of anxiety, separately for women and separately for men.

The statistical analysis has been carried out with DELL STATISTICA (data analysis software system) version 13 Dell Inc (2016, software.dell.com). A value of *p* ≤ 0.05 was considered statistically significant.

## 3. Results

The results obtained from the first and second part of the questionnaire concerning anxiety as a state and as a trait showed high level of anxiety as a state in 51.19% of the students and as a trait in 32.14% and low level in 19.05% and 41.67%, respectively (Table 1).

Result analysis showed high level of anxiety as a state in 47.62% females and 61.90% males, while anxiety as a trait was high in 31.75% and 33.33%, respectively. Low anxiety as a state level was noted in 20.63% females and in 14.29% males, and for anxiety as a trait, these values were 41.27% and 42.86%, respectively (Table 1). Statistical significance was observed between groups of females with low and high anxiety as a state (*p*=0.02) and between males with low and high (*p*=0.004) as well as normal and high (*p*=0.01) anxiety as a state.

The obtained results showed minimal and maximal values to be 24 and 71, respectively, for the STAI-1 scale, and 24 and 57, respectively, for the STAI-2 scale. Average values were 40.55 (STAI-1) and 41.75 (STAI-2) (Table 2). Statistical analysis has not revealed significance between anxiety as a state and as a trait.

The answers obtained from the I and II part of the questionnaire evaluating anxiety as a state and as a trait show that in the studied group of 35 students who presented with low anxiety as a trait, 14 of them (40.00%) presented with a low level of anxiety as a state, while within 27 students whose anxiety as a trait was classified as high, 21 (77.78%) presented also with high level of anxiety as a state. Statistical analysis displayed statistical relationship between the studied state and trait (*p*=0.0001) (Table 3).

## 4. Discussion

Obligatory curriculum of the study of dentistry includes theoretical as well as practical training. In Poland, the guidelines developed by the Ministry of Science and Higher Education provide for 85% of classes to be carried out as clinical training involving the treatment of patients.

Dental students are exposed to stressing situations during clinical classes as part of their curriculum [17]. However, publications concerning this issue are scarce. Moreover, there is no information on the number of Polish students seeking help from a psychiatrist or a psychologist to cope with such problems.

Practical classes in pediatric dentistry are preceded by theoretical lectures and seminars, phantom classes, and patient-doctor communication instruction classes. The students also do summer training which is supposed to prepare them for the work with patients. Practical classes as a part of the third year curriculum are one of the first clinical classes in the dental studies and involve noninvasive procedures, mostly prophylactic, to be performed by the students. Examination of a young patient is always done under the supervision of experienced doctors; therefore, the students are able to consult their supervisor on every stage of clinical management. The presence of a parent or legal guardian is also accepted. The students not only treat but also educate young patients and their parents on proper diet, oral hygiene, prevention of caries, and oral mucosa inflammation.

In the students' opinion, a direct relationship with a patient causes additional difficulties comparing to phantom classes simulations, as not only theoretical knowledge needs to be verified practically but also own emotions have to be tamed. Such reactions are likely to appear during clinical classes in pediatric dentistry when undertaking prophylactic and therapeutic actions on patients in the developmental age.

It is commonly known that a new experience, like performing a procedure for the first time, may cause significant anxiety and stress both in adepts of medical art and experienced doctors with many years of practice [9].

Supposingly, anxiety level varies and depends, among others, on personality and temperament of an operator [13]. Therefore, assessment of anxiety as a trait in dental students shows them being or not being ready for delivering dental care during clinical classes to a patient in the developmental age.

Kaczmarek et al. [18] researched anxiety in 53 fourth year dental students before they were to start dental treatment of children. The study showed that half of the students present with moderate anxiety, both as a trait (54.3%) and state (55.0%). The authors were mainly focused on the students' stress management, based on the COPE scale-questionnaire. The most common strategy to deal with stress was “positive perception of the world and development,” and the least common is “the use of stimulants.” Some students chose “negation/ignoring the problem” as a way to cope with stress [18].

Babar et al. [19] studied 529 students of dentistry studying on the years from first to fifth in one of the private colleges in Malaysia. The Dental Environment Stress (DES) questionnaire was applied to evaluate stress level. Interestingly, the fear of failure in a course was the greatest stressor, as described by the students of all years.

Davidovich et al. [20] researched self-reported stress of general practitioners, dental students, and specialists in pediatric dentistry during the performance of different procedures in pediatric patients. The authors revealed that for the experienced dentists, both the general practitioners and the specialists, injection of local anesthesia to an anxious child was the most stressful procedure. However, dental students reported placing a rubber dam as such a challenge.

Studies by various authors point to different types of stimulants used by the students experiencing mental or emotional tension and stress [4, 22, 23, 21]. Sniatala et al. [21] performed a survey among 187 Polish students of a medical college including the students of dentistry (78.07%) and oral hygiene (21.93%). The study has shown that 12.30% of the respondents referred to cigarette smoking and 10.70% to alcohol consumption in stressful situations. Ne'Eman-Haviv and Bonny-Noach [22] performed a questionnaire study in 814 undergraduate students concerning association between the use of alcohol, tobacco, cannabis, and medical and nonmedical prescription stimulants (MNPS) and cognitive test anxiety (CTA). The study has shown that CTA was higher among users of MNPS than among students who did not use such substances. This is in agreement with Erdal et al.'s results [23] of the research on the students of Gaziosmanpasa University in Turkey who smoked cigarettes, since thirty percent of them admitted they smoke to reduce stress. Moreover, it must be emphasized that the risk of addiction, both somatic and psychical, to various chemical substances is greater if they are applied to reduce stress [24]. Needless to say, addictive use during medical school may affect students' professional and personal lives [25].

On the other hand, the dental environment-induced stress could negatively affect well-being of students [26]. They might be unable to interact with the patients or to continue studying and working. Therefore, due to elevated level of stress their career options can be reduced.

At present, there are different methods available to fight stress. Shankarapillai et al. [27] described an advantageous effect of yoga on reducing state-trait anxiety level in students of dentistry. Moreover, deep breathing, progressive muscle relaxation, or hypnosis are also recommended [10].

Pereira et al. [28] presented data concerning an elective course named “Strategies of Coping with Professional Stress” which was offered to medical students of a midwest public Brazilian university. Interestingly, majority of students (67.1%) answered that their stress symptoms decreased by the end of the course. Moreover, it must be emphasized that the students were eager to participate in the electives what proves their great interest in the issue and their belief that there is a need to be rightly prepared to cope with stress, both during the studies and in future work.

Piazza-Waggoner et al. [9] carried out research on 26 second year students of dentistry to assess their anxiety during their first treatment of a pediatric patient. Their initial assessment was done using a Visual Analog Scale (VAS) and STAI and COPE (Coping Orientation with Problems Experienced) questionnaires. The students were divided into two groups: anxiety management group and attention control group. In the first group, the participants received training on relaxation methods, including deep breathing and progressive muscle relaxation. Additionally, they were given a casette with an instruction on how to perform these exercises and were supposed to listen to it while doing the exercises at least once a day. The control group attended a lecture on the relationship between anxiety, stress, and health and received a casette with an ocean waves sound recorded, without any instruction. The authors showed that anxiety as a trait (STAI Trait Score) in the students ranged between 24 and 59 (average 37.5; SD = 9.8), while anxiety as a state (STAI State Score) between 20 and 54 (average 35.0; SD = 10.1).

Therefore, it seems necessary to carry out exercises to reduce the anxiety level. The students may practice such relaxation themselves or during breaks between classes. These procedures are costless and may bring only positive effect.

Finally, some strength and limitations of the present study should be described. On the one hand, the strength of the study was that all 3^rd^ year Polish dental students, who studied at the Poznan University of Medical Sciences during the research, were examined. However, the sample is not representative of the population of the entire Polish third year dental students since they were from one university. Therefore, it is suggested that such studies should be carried out, using the same methodology, in most dental schools in the country. Moreover, it seems necessary to continue the research to compare the results with those obtained for the same students in the following years. It could be interesting to perform such follow-up to observe the changes in the level of anxiety in individuals. Another limitation is that most of the researchers did not use the same methodology but rather different questionnaires. Therefore, it is difficult to compare the results with those of other authors. However, main strength of the present study is to highlight a state of anxiety in dental students that could have a detrimental effect in performance and learning of dental techniques.

Addition, our data were based on self-reported measures (i.e., questionnaire), which might be influenced by mono-method bias. But future survey may therefore benefit from replicating our findings in longitudinal studies.

However, the implications derived from the present research appear useful since they might show what level of anxiety dental students represent. Therefore, special programs concerning stress fighting and relaxation may be introduced at the universities [29]. It needs to be emphasized that young adults form a population with specific characteristics that have to be taken into account in the management of occupational risks. The implications derived from this study might also be helpful at the individual level for mental health service purposes if any person may need such support.

## 5. Conclusions

The results show that the anxiety level during clinical classes is relatively high in the studied population of students. Preparing the students to cope with stress resulting from treating the patients seems to be of importance. Such programmes should be implemented before the start of practical clinical classes. The acquired knowledge will be useful in further professional career.

## Figures and Tables

**Table 1:** Anxiety as a state (STAI-1) and anxiety as a trait (STAI-2).

		Females	Males	Total	^*∗*^ *p* value
					Females vs males
*STAI-1*					
Low	N	13	3	16	ns
	%	20.63	14.29	19.05	
Normal	N	20	5	25	ns
	%	31.75	23.81	26.76	
High	N	30	13	43	ns
	%	47.62	61.90	51.19	
Total		63	21	84	
^*∗*^ *p* value	Low vs normal	ns	ns		
	Normal vs high	ns	*p*=0.01		
	Low vs high	*p*=0.02	*p*=0.004		

*STAI-2*					
Low	N	26	9	35	ns
	%	41.27	42.86	41.67	
Normal	N	17	5	22	ns
	%	26.98	23.81	26.19	
High	N	20	7	27	ns
	%	31.75	33.33	32.14	
Total		63	21	84		
^*∗*^ *p* value	Low vs normal	ns	ns		
	Normal vs high	ns	ns		
	Low vs high	ns	ns		

^*∗*^Difference test between two proportions.

**Table 2:** Average, minimal, and maximal STAI values.

Scale	N	X ± SD	Min–Max
STAI-2	84	41.75 ± 7.63	24.00–57.00
STAI-1	84	40.55 ± 10.54	24.00–71.00
^*∗*^ *p* value	ns		

^*∗*^Wilcoxon matched-pair test.

**Table 3:** Relationship between anxiety as a state (STAI-1) and as a trait (STAI-2).

Anxiety level		STAI-1		STAI-2			Total	*p* value		
				1	2	3		1 vs 2	2 vs 3	1 vs 3
Low		1	N	14	2	0	16	*p*=0.04	ns	*p*=0.005
			%	40.00	9.09	0.00				
Normal		2	N	11	8	6	25	ns	ns	ns
			%	31.43	36.36	22.22				
High		3	N	10	12	21	43	*p*=0.01	*p*=0.02	*p*=0.0001
			%	28.57	54.55	77.78				
Total			N	35	22	27	84			
			%	41.67	26.19	32.14				
^*∗*^ *p* value	Low vs normal	ns	*p*=0.03	*p*=0.01					
	Normal vs high	ns	ns	*p*=0.0001					
	Low vs high	ns	*p*=0.001	*p*=0.0001					

^*∗*^Difference test between two proportions.

## Data Availability

All data are available on request.
